# Supplementary material for: Risk factors for osteoporosis in elderly patients with type 2 diabetes: A protocol for systematic review and meta-analysis
Source: PLoS One. 2025 Feb 27;20(2):e0319602. doi: 10.1371/journal.pone.0319602 (PMC11867315; doi:10.1371/journal.pone.0319602)
Supplement: S3 File — (DOCX) [file pone.0319602.s004.docx]

**S2 File Search strategy**

| **PubMed** | |
| --- | --- |
| # | Query |
| #1 | "Aged"[Mesh] OR (Aged[Title/Abstract]) OR (Elderly[Title/Abstract]) |
| #2 | “Diabetes Mellitus”[Mesh] |
| #3 | “Diabetes Mellitus, Type 2”[Title/Abstract] OR “Diabetes Mellitus, Adult-Onset”[Title/Abstract] OR “Adult-Onset Diabetes Mellitus”[Title/Abstract] OR “Diabetes Mellitus, Adult Onset”[Title/Abstract] OR “Diabetes Mellitus, Ketosis-Resistant”[Title/Abstract] OR “Diabetes Mellitus, Ketosis Resistant”[Title/Abstract] OR “Ketosis-Resistant Diabetes Mellitus”[Title/Abstract] OR “Diabetes Mellitus, Non Insulin Dependent”[Title/Abstract] OR “Diabetes Mellitus, Non-Insulin-Dependent”[Title/Abstract] OR “Non-Insulin-Dependent Diabetes Mellitus”[Title/Abstract] OR “Diabetes Mellitus, Stable”[Title/Abstract] OR “Stable Diabetes Mellitus”[Title/Abstract] OR “Diabetes Mellitus, Type II”[Title/Abstract] OR “NIDDM”[Title/Abstract] OR “Diabetes Mellitus, Noninsulin Dependent”[Title/Abstract] OR “Diabetes Mellitus, Maturity-Onset”[Title/Abstract] OR “Diabetes Mellitus, Maturity Onset”[Title/Abstract] OR “Maturity-Onset Diabetes Mellitus”[Title/Abstract] OR “Maturity Onset Diabetes Mellitus”[Title/Abstract] OR “MODY”[Title/Abstract] OR “Diabetes Mellitus, Slow-Onset”[Title/Abstract] OR “Diabetes Mellitus, Slow Onset”[Title/Abstract] OR “Slow-Onset Diabetes Mellitus”[Title/Abstract] OR “Type 2 Diabetes Mellitus”[Title/Abstract] OR “Noninsulin-Dependent Diabetes Mellitus”[Title/Abstract] OR “Noninsulin Dependent Diabetes Mellitus”[Title/Abstract] OR “Maturity-Onset Diabetes”[Title/Abstract] OR “Diabetes, Maturity-Onset”[Title/Abstract] OR “Maturity Onset Diabetes”[Title/Abstract] OR “Type 2 Diabetes”[Title/Abstract] OR “Diabetes, Type 2”[Title/Abstract] OR “Diabetes Mellitus, Noninsulin-Dependent”[Title/Abstract] OR  “Type 1 Diabetes”[Title/Abstract] OR “Diabetes, Type 1”[Title/Abstract] OR “Diabetes Mellitus, Insulin-Dependent”[Title/Abstract] OR “Diabetes Mellitus, Insulin Dependent”[Title/Abstract] OR “Insulin-Dependent Diabetes Mellitus”[Title/Abstract] OR “Diabetes Mellitus, Juvenile-Onset”[Title/Abstract] OR “Diabetes Mellitus, Juvenile Onset”[Title/Abstract] OR “Juvenile-Onset Diabetes Mellitus”[Title/Abstract] OR “IDDM”[Title/Abstract] OR “Diabetes Mellitus, Type I”[Title/Abstract] OR “Diabetes Mellitus, Sudden-Onset”[Title/Abstract] OR “Diabetes Mellitus, Sudden Onset”[Title/Abstract] OR “Sudden-Onset Diabetes Mellitus”[Title/Abstract] OR “Type 1 Diabetes Mellitus”[Title/Abstract] OR “Diabetes Mellitus, Insulin-Dependent, 1”[Title/Abstract] OR “Insulin-Dependent Diabetes Mellitus 1”[Title/Abstract] OR “Insulin Dependent Diabetes Mellitus 1”[Title/Abstract] OR “Juvenile-Onset Diabetes”[Title/Abstract] OR “Diabetes, Juvenile-Onset”[Title/Abstract] OR “Juvenile Onset Diabetes”[Title/Abstract] OR “Diabetes, Autoimmune”[Title/Abstract] OR “Autoimmune Diabetes”[Title/Abstract] OR “Diabetes Mellitus, Brittle”[Title/Abstract] OR “Brittle Diabetes Mellitus”[Title/Abstract] OR “Diabetes Mellitus, Ketosis-Prone”[Title/Abstract] OR “Diabetes Mellitus, Ketosis Prone”[Title/Abstract] OR “Ketosis-Prone Diabetes Mellitus”[Title/Abstract] |
| #4 | #2 OR #3 |
| #5 | “Osteoporosis”[Mesh] |
| #6 | “Osteoporoses”[Title/Abstract] OR Osteoporosis, Age-Related[Title/Abstract] OR “Osteoporosis, Age Related”[Title/Abstract] OR “Age-Related Osteoporosis”[Title/Abstract] OR “Age-Related Osteoporoses”[Title/Abstract] OR “Age Related Osteoporosis”[Title/Abstract] OR ““Osteoporoses, Age-Related”[Title/Abstract] OR “Bone Loss, Age-Related”[Title/Abstract] OR  “Age-Related Bone Loss”[Title/Abstract] OR “Age-Related Bone Losses”[Title/Abstract] OR “Bone Loss, Age Related”[Title/Abstract] OR “Bone Losses, Age-Related”[Title/Abstract] OR “Osteoporosis, Senile”[Title/Abstract] OR “Osteoporoses, Senile”[Title/Abstract] OR “Senile Osteoporoses”[Title/Abstract] OR “Senile Osteoporosis”[Title/Abstract] OR “Osteoporosis, Involutional”[Title/Abstract] OR “Osteoporosis, Post-Traumatic”[Title/Abstract] OR “Osteoporosis, Post Traumatic”[Title/Abstract] OR “Post-Traumatic Osteoporoses”[Title/Abstract] OR “Post-Traumatic Osteoporosis”[Title/Abstract] |
| #7 | “Fractures, Bone”[Mesh] |
| #8 | “Bone Fracture”[Title/Abstract] OR “Fracture, Bone”[Title/Abstract] OR “Bone Fractures”[Title/Abstract] OR “Broken Bones”[Title/Abstract] OR “Bone, Broken”[Title/Abstract] OR “Bones, Broken”[Title/Abstract] OR “Broken Bone”[Title/Abstract] OR “Spiral Fractures”[Title/Abstract] OR “Fracture, Spiral”[Title/Abstract] OR “Fractures, Spiral”[Title/Abstract] OR “Spiral Fracture”[Title/Abstract] OR “Torsion Fractures”[Title/Abstract] OR “Fractures, Torsion”[Title/Abstract] OR “Fracture, Torsion”[Title/Abstract] OR “Torsion Fracture”[Title/Abstract] |
| #9 | “Accidental Falls”[Mesh] |
| #10 | “Falls, Accidental”[Title/Abstract] OR “Accidental Fall”[Title/Abstract] OR “Fall, Accidental”[Title/Abstract] OR “Falling”[Title/Abstract] OR “Falls”[Title/Abstract] OR “Slip and Fall”[Title/Abstract] OR “Fall and Slip”[Title/Abstract] |
| #11 | #5 OR #6 OR #7 OR #8 OR #9 OR #10 |
| #12 | “Risk Factors”[Mesh] |
| #13 | (“Risk Factors”[Title/Abstract] OR “Factor, Risk”[Title/Abstract] OR “Risk Factor”[Title/Abstract] OR “Population at Risk”[Title/Abstract] OR “Populations at Risk”[Title/Abstract] OR “Risk Scores”[Title/Abstract] OR “Risk Score”[Title/Abstract] OR “Score, Risk”[Title/Abstract] OR “Risk Factor Scores”[Title/Abstract] OR “Risk Factor Score”[Title/Abstract] OR “Score, Risk Factor”[Title/Abstract] OR “Health Correlates”[Title/Abstract] OR “Correlates, Health”[Title/Abstract] OR “Social Risk Factors”[Title/Abstract] OR “Factor, Social Risk”[Title/Abstract] OR “Factors, Social Risk”[Title/Abstract] OR “Risk Factor, Social”[Title/Abstract] OR “Risk Factors, Social”[Title/Abstract] OR “Social Risk Factor”[Title/Abstract]) |
| #14 | #1 AND #4 AND #11 AND 11 |
| **Web of Science** | |
| #1 | TS=(“Diabetes Mellitus, Type 2” OR “Diabetes Mellitus, Adult-Onset” OR “Adult-Onset Diabetes Mellitus” OR “Diabetes Mellitus, Adult Onset” OR “Diabetes Mellitus, Ketosis-Resistant” OR “Diabetes Mellitus, Ketosis Resistant” OR “Ketosis-Resistant Diabetes Mellitus” OR “Diabetes Mellitus, Non Insulin Dependent” OR “Diabetes Mellitus, Non-Insulin-Dependent” OR “Non-Insulin-Dependent Diabetes Mellitus” OR “Diabetes Mellitus, Stable” OR “Stable Diabetes Mellitus” OR “Diabetes Mellitus, Type II” OR “NIDDM” OR “Diabetes Mellitus, Noninsulin Dependent” OR “Diabetes Mellitus, Maturity-Onset” OR “Diabetes Mellitus, Maturity Onset” OR “Maturity-Onset Diabetes Mellitus” OR “Maturity Onset Diabetes Mellitus” OR “MODY” OR “Diabetes Mellitus, Slow-Onset” OR “Diabetes Mellitus, Slow Onset” OR “Slow-Onset Diabetes Mellitus” OR “Type 2 Diabetes Mellitus” OR “Noninsulin-Dependent Diabetes Mellitus” OR “Noninsulin Dependent Diabetes Mellitus” OR “Maturity-Onset Diabetes” OR “Diabetes, Maturity-Onset” OR “Maturity Onset Diabetes” OR “Type 2 Diabetes” OR “Diabetes, Type 2” OR “Diabetes Mellitus, Noninsulin-Dependent” OR “Type 1 Diabetes” OR “Diabetes, Type 1” OR “Diabetes Mellitus, Insulin-Dependent” OR “Diabetes Mellitus, Insulin Dependent” OR “Insulin-Dependent Diabetes Mellitus” OR “Diabetes Mellitus, Juvenile-Onset” OR “Diabetes Mellitus, Juvenile Onset” OR “Juvenile-Onset Diabetes Mellitus” OR “IDDM” OR “Diabetes Mellitus, Type I” OR “Diabetes Mellitus, Sudden-Onset” OR “Diabetes Mellitus, Sudden Onset” OR “Sudden-Onset Diabetes Mellitus” OR “Type 1 Diabetes Mellitus” OR “Diabetes Mellitus, Insulin-Dependent, 1” OR “Insulin-Dependent Diabetes Mellitus 1” OR “Insulin Dependent Diabetes Mellitus 1” OR “Juvenile-Onset Diabetes” OR “Diabetes, Juvenile-Onset” OR “Juvenile Onset Diabetes” OR “Diabetes, Autoimmune” OR “Autoimmune Diabetes” OR “Diabetes Mellitus, Brittle” OR “Brittle Diabetes Mellitus” OR “Diabetes Mellitus, Ketosis-Prone” OR “Diabetes Mellitus, Ketosis Prone” OR “Ketosis-Prone Diabetes Mellitus”) |
| #2 | TS= (age* OR elder* OR old* OR senior*) |
| #3 | TS= (“Osteoporoses” OR Osteoporosis, Age-Related OR “Osteoporosis, Age Related” OR “Age-Related Osteoporosis” OR “Age-Related Osteoporoses” OR “Age Related Osteoporosis” OR ““Osteoporoses, Age-Related” OR “Bone Loss, Age-Related” OR  “Age-Related Bone Loss” OR “Age-Related Bone Losses” OR “Bone Loss, Age Related” OR “Bone Losses, Age-Related” OR “Osteoporosis, Senile” OR “Osteoporoses, Senile” OR “Senile Osteoporoses” OR “Senile Osteoporosis” OR “Osteoporosis, Involutional” OR “Osteoporosis, Post-Traumatic” OR “Osteoporosis, Post Traumatic” OR “Post-Traumatic Osteoporoses” OR “Post-Traumatic Osteoporosis”) |
| #4 | TS= (“Bone Fracture” OR “Fracture, Bone” OR “Bone Fractures” OR “Broken Bones” OR “Bone, Broken” OR “Bones, Broken” OR “Broken Bone” OR “Spiral Fractures” OR “Fracture, Spiral” OR “Fractures, Spiral” OR “Spiral Fracture” OR “Torsion Fractures” OR “Fractures, Torsion” OR “Fracture, Torsion” OR “Torsion Fracture”) |
| #5 | TS= (“Falls, Accidental” OR “Accidental Fall” OR “Fall, Accidental” OR “Falling” OR “Falls” OR “Slip and Fall” OR “Fall and Slip”) |
| #6 | TS= (“Risk Factors” OR “Factor, Risk” OR “Risk Factor” OR “Population at Risk” OR “Populations at Risk” OR “Risk Scores” OR “Risk Score” OR “Score, Risk” OR “Risk Factor Scores” OR “Risk Factor Score” OR “Score, Risk Factor” OR “Health Correlates” OR “Correlates, Health” OR “Social Risk Factors” OR “Factor, Social Risk” OR “Factors, Social Risk” OR “Risk Factor, Social” OR “Risk Factors, Social” OR “Social Risk Factor”) |
| #7 | #3 OR #4 OR #5 |
| #8 | #1 AND #2 #6 AND #7 |
| **CINAHL** | |
| #1 | TX(“Diabetes Mellitus, Type 2” OR “Diabetes Mellitus, Adult-Onset” OR “Adult-Onset Diabetes Mellitus” OR “Diabetes Mellitus, Adult Onset” OR “Diabetes Mellitus, Ketosis-Resistant” OR “Diabetes Mellitus, Ketosis Resistant” OR “Ketosis-Resistant Diabetes Mellitus” OR “Diabetes Mellitus, Non Insulin Dependent” OR “Diabetes Mellitus, Non-Insulin-Dependent” OR “Non-Insulin-Dependent Diabetes Mellitus” OR “Diabetes Mellitus, Stable” OR “Stable Diabetes Mellitus” OR “Diabetes Mellitus, Type II” OR “NIDDM” OR “Diabetes Mellitus, Noninsulin Dependent” OR “Diabetes Mellitus, Maturity-Onset” OR “Diabetes Mellitus, Maturity Onset” OR “Maturity-Onset Diabetes Mellitus” OR “Maturity Onset Diabetes Mellitus” OR “MODY” OR “Diabetes Mellitus, Slow-Onset” OR “Diabetes Mellitus, Slow Onset” OR “Slow-Onset Diabetes Mellitus” OR “Type 2 Diabetes Mellitus” OR “Noninsulin-Dependent Diabetes Mellitus” OR “Noninsulin Dependent Diabetes Mellitus” OR “Maturity-Onset Diabetes” OR “Diabetes, Maturity-Onset” OR “Maturity Onset Diabetes” OR “Type 2 Diabetes” OR “Diabetes, Type 2” OR “Diabetes Mellitus, Noninsulin-Dependent” OR “Type 1 Diabetes” OR “Diabetes, Type 1” OR “Diabetes Mellitus, Insulin-Dependent” OR “Diabetes Mellitus, Insulin Dependent” OR “Insulin-Dependent Diabetes Mellitus” OR “Diabetes Mellitus, Juvenile-Onset” OR “Diabetes Mellitus, Juvenile Onset” OR “Juvenile-Onset Diabetes Mellitus” OR “IDDM” OR “Diabetes Mellitus, Type I” OR “Diabetes Mellitus, Sudden-Onset” OR “Diabetes Mellitus, Sudden Onset” OR “Sudden-Onset Diabetes Mellitus” OR “Type 1 Diabetes Mellitus” OR “Diabetes Mellitus, Insulin-Dependent, 1” OR “Insulin-Dependent Diabetes Mellitus 1” OR “Insulin Dependent Diabetes Mellitus 1” OR “Juvenile-Onset Diabetes” OR “Diabetes, Juvenile-Onset” OR “Juvenile Onset Diabetes” OR “Diabetes, Autoimmune” OR “Autoimmune Diabetes” OR “Diabetes Mellitus, Brittle” OR “Brittle Diabetes Mellitus” OR “Diabetes Mellitus, Ketosis-Prone” OR “Diabetes Mellitus, Ketosis Prone” OR “Ketosis-Prone Diabetes Mellitus”) |
| #2 | TX(age* OR elder* OR old* OR senior*) |
| #3 | TX(“Osteoporoses” OR Osteoporosis, Age-Related OR “Osteoporosis, Age Related” OR “Age-Related Osteoporosis” OR “Age-Related Osteoporoses” OR “Age Related Osteoporosis” OR ““Osteoporoses, Age-Related” OR “Bone Loss, Age-Related” OR  “Age-Related Bone Loss” OR “Age-Related Bone Losses” OR “Bone Loss, Age Related” OR “Bone Losses, Age-Related” OR “Osteoporosis, Senile” OR “Osteoporoses, Senile” OR “Senile Osteoporoses” OR “Senile Osteoporosis” OR “Osteoporosis, Involutional” OR “Osteoporosis, Post-Traumatic” OR “Osteoporosis, Post Traumatic” OR “Post-Traumatic Osteoporoses” OR “Post-Traumatic Osteoporosis”) |
| #4 | TX(“Bone Fracture” OR “Fracture, Bone” OR “Bone Fractures” OR “Broken Bones” OR “Bone, Broken” OR “Bones, Broken” OR “Broken Bone” OR “Spiral Fractures” OR “Fracture, Spiral” OR “Fractures, Spiral” OR “Spiral Fracture” OR “Torsion Fractures” OR “Fractures, Torsion” OR “Fracture, Torsion” OR “Torsion Fracture”) |
| #5 | TX (“Falls, Accidental” OR “Accidental Fall” OR “Fall, Accidental” OR “Falling” OR “Falls” OR “Slip and Fall” OR “Fall and Slip”) |
| #6 | TX(“Risk Factors” OR “Factor, Risk” OR “Risk Factor” OR “Population at Risk” OR “Populations at Risk” OR “Risk Scores” OR “Risk Score” OR “Score, Risk” OR “Risk Factor Scores” OR “Risk Factor Score” OR “Score, Risk Factor” OR “Health Correlates” OR “Correlates, Health” OR “Social Risk Factors” OR “Factor, Social Risk” OR “Factors, Social Risk” OR “Risk Factor, Social” OR “Risk Factors, Social” OR “Social Risk Factor”) |
| #7 | #3 OR #4 OR #5 |
| #8 | #1 AND #2 #6 AND #7 |
| **Cochrane Library** | |
| #1 | (“Diabetes Mellitus, Type 2” OR “Diabetes Mellitus, Adult-Onset” OR “Adult-Onset Diabetes Mellitus” OR “Diabetes Mellitus, Adult Onset” OR “Diabetes Mellitus, Ketosis-Resistant” OR “Diabetes Mellitus, Ketosis Resistant” OR “Ketosis-Resistant Diabetes Mellitus” OR “Diabetes Mellitus, Non Insulin Dependent” OR “Diabetes Mellitus, Non-Insulin-Dependent” OR “Non-Insulin-Dependent Diabetes Mellitus” OR “Diabetes Mellitus, Stable” OR “Stable Diabetes Mellitus” OR “Diabetes Mellitus, Type II” OR “NIDDM” OR “Diabetes Mellitus, Noninsulin Dependent” OR “Diabetes Mellitus, Maturity-Onset” OR “Diabetes Mellitus, Maturity Onset” OR “Maturity-Onset Diabetes Mellitus” OR “Maturity Onset Diabetes Mellitus” OR “MODY” OR “Diabetes Mellitus, Slow-Onset” OR “Diabetes Mellitus, Slow Onset” OR “Slow-Onset Diabetes Mellitus” OR “Type 2 Diabetes Mellitus” OR “Noninsulin-Dependent Diabetes Mellitus” OR “Noninsulin Dependent Diabetes Mellitus” OR “Maturity-Onset Diabetes” OR “Diabetes, Maturity-Onset” OR “Maturity Onset Diabetes” OR “Type 2 Diabetes” OR “Diabetes, Type 2” OR “Diabetes Mellitus, Noninsulin-Dependent” OR “Type 1 Diabetes” OR “Diabetes, Type 1” OR “Diabetes Mellitus, Insulin-Dependent” OR “Diabetes Mellitus, Insulin Dependent” OR “Insulin-Dependent Diabetes Mellitus” OR “Diabetes Mellitus, Juvenile-Onset” OR “Diabetes Mellitus, Juvenile Onset” OR “Juvenile-Onset Diabetes Mellitus” OR “IDDM” OR “Diabetes Mellitus, Type I” OR “Diabetes Mellitus, Sudden-Onset” OR “Diabetes Mellitus, Sudden Onset” OR “Sudden-Onset Diabetes Mellitus” OR “Type 1 Diabetes Mellitus” OR “Diabetes Mellitus, Insulin-Dependent, 1” OR “Insulin-Dependent Diabetes Mellitus 1” OR “Insulin Dependent Diabetes Mellitus 1” OR “Juvenile-Onset Diabetes” OR “Diabetes, Juvenile-Onset” OR “Juvenile Onset Diabetes” OR “Diabetes, Autoimmune” OR “Autoimmune Diabetes” OR “Diabetes Mellitus, Brittle” OR “Brittle Diabetes Mellitus” OR “Diabetes Mellitus, Ketosis-Prone” OR “Diabetes Mellitus, Ketosis Prone” OR “Ketosis-Prone Diabetes Mellitus”):ti,ab,kw |
| #2 | (age* OR elder* OR old* OR senior*):ti,ab,kw |
| #3 | (“Osteoporoses” OR Osteoporosis, Age-Related OR “Osteoporosis, Age Related” OR “Age-Related Osteoporosis” OR “Age-Related Osteoporoses” OR “Age Related Osteoporosis” OR ““Osteoporoses, Age-Related” OR “Bone Loss, Age-Related” OR  “Age-Related Bone Loss” OR “Age-Related Bone Losses” OR “Bone Loss, Age Related” OR “Bone Losses, Age-Related” OR “Osteoporosis, Senile” OR “Osteoporoses, Senile” OR “Senile Osteoporoses” OR “Senile Osteoporosis” OR “Osteoporosis, Involutional” OR “Osteoporosis, Post-Traumatic” OR “Osteoporosis, Post Traumatic” OR “Post-Traumatic Osteoporoses” OR “Post-Traumatic Osteoporosis”):ti,ab,kw |
| #4 | (“Bone Fracture” OR “Fracture, Bone” OR “Bone Fractures” OR “Broken Bones” OR “Bone, Broken” OR “Bones, Broken” OR “Broken Bone” OR “Spiral Fractures” OR “Fracture, Spiral” OR “Fractures, Spiral” OR “Spiral Fracture” OR “Torsion Fractures” OR “Fractures, Torsion” OR “Fracture, Torsion” OR “Torsion Fracture”):ti,ab,kw |
| #5 | (“Falls, Accidental” OR “Accidental Fall” OR “Fall, Accidental” OR “Falling” OR “Falls” OR “Slip and Fall” OR “Fall and Slip”) |
| #6 | (“Risk Factors” OR “Factor, Risk” OR “Risk Factor” OR “Population at Risk” OR “Populations at Risk” OR “Risk Scores” OR “Risk Score” OR “Score, Risk” OR “Risk Factor Scores” OR “Risk Factor Score” OR “Score, Risk Factor” OR “Health Correlates” OR “Correlates, Health” OR “Social Risk Factors” OR “Factor, Social Risk” OR “Factors, Social Risk” OR “Risk Factor, Social” OR “Risk Factors, Social” OR “Social Risk Factor”):ti,ab,kw |
| #7 | #3 OR #4 OR #5 |
| #8 | #1 AND #2 #6 AND #7 |
| **EMBASE** | |
| #1 | (“Diabetes Mellitus, Type 2” OR “Diabetes Mellitus, Adult-Onset” OR “Adult-Onset Diabetes Mellitus” OR “Diabetes Mellitus, Adult Onset” OR “Diabetes Mellitus, Ketosis-Resistant” OR “Diabetes Mellitus, Ketosis Resistant” OR “Ketosis-Resistant Diabetes Mellitus” OR “Diabetes Mellitus, Non Insulin Dependent” OR “Diabetes Mellitus, Non-Insulin-Dependent” OR “Non-Insulin-Dependent Diabetes Mellitus” OR “Diabetes Mellitus, Stable” OR “Stable Diabetes Mellitus” OR “Diabetes Mellitus, Type II” OR “NIDDM” OR “Diabetes Mellitus, Noninsulin Dependent” OR “Diabetes Mellitus, Maturity-Onset” OR “Diabetes Mellitus, Maturity Onset” OR “Maturity-Onset Diabetes Mellitus” OR “Maturity Onset Diabetes Mellitus” OR “MODY” OR “Diabetes Mellitus, Slow-Onset” OR “Diabetes Mellitus, Slow Onset” OR “Slow-Onset Diabetes Mellitus” OR “Type 2 Diabetes Mellitus” OR “Noninsulin-Dependent Diabetes Mellitus” OR “Noninsulin Dependent Diabetes Mellitus” OR “Maturity-Onset Diabetes” OR “Diabetes, Maturity-Onset” OR “Maturity Onset Diabetes” OR “Type 2 Diabetes” OR “Diabetes, Type 2” OR “Diabetes Mellitus, Noninsulin-Dependent” OR “Type 1 Diabetes” OR “Diabetes, Type 1” OR “Diabetes Mellitus, Insulin-Dependent” OR “Diabetes Mellitus, Insulin Dependent” OR “Insulin-Dependent Diabetes Mellitus” OR “Diabetes Mellitus, Juvenile-Onset” OR “Diabetes Mellitus, Juvenile Onset” OR “Juvenile-Onset Diabetes Mellitus” OR “IDDM” OR “Diabetes Mellitus, Type I” OR “Diabetes Mellitus, Sudden-Onset” OR “Diabetes Mellitus, Sudden Onset” OR “Sudden-Onset Diabetes Mellitus” OR “Type 1 Diabetes Mellitus” OR “Diabetes Mellitus, Insulin-Dependent, 1” OR “Insulin-Dependent Diabetes Mellitus 1” OR “Insulin Dependent Diabetes Mellitus 1” OR “Juvenile-Onset Diabetes” OR “Diabetes, Juvenile-Onset” OR “Juvenile Onset Diabetes” OR “Diabetes, Autoimmune” OR “Autoimmune Diabetes” OR “Diabetes Mellitus, Brittle” OR “Brittle Diabetes Mellitus” OR “Diabetes Mellitus, Ketosis-Prone” OR “Diabetes Mellitus, Ketosis Prone” OR “Ketosis-Prone Diabetes Mellitus”):ti,ab |
| #2 | (age* OR elder* OR old* OR senior*):ti,ab |
| #3 | (“Osteoporoses” OR Osteoporosis, Age-Related OR “Osteoporosis, Age Related” OR “Age-Related Osteoporosis” OR “Age-Related Osteoporoses” OR “Age Related Osteoporosis” OR ““Osteoporoses, Age-Related” OR “Bone Loss, Age-Related” OR  “Age-Related Bone Loss” OR “Age-Related Bone Losses” OR “Bone Loss, Age Related” OR “Bone Losses, Age-Related” OR “Osteoporosis, Senile” OR “Osteoporoses, Senile” OR “Senile Osteoporoses” OR “Senile Osteoporosis” OR “Osteoporosis, Involutional” OR “Osteoporosis, Post-Traumatic” OR “Osteoporosis, Post Traumatic” OR “Post-Traumatic Osteoporoses” OR “Post-Traumatic Osteoporosis”):ti,ab |
| #4 | (“Bone Fracture” OR “Fracture, Bone” OR “Bone Fractures” OR “Broken Bones” OR “Bone, Broken” OR “Bones, Broken” OR “Broken Bone” OR “Spiral Fractures” OR “Fracture, Spiral” OR “Fractures, Spiral” OR “Spiral Fracture” OR “Torsion Fractures” OR “Fractures, Torsion” OR “Fracture, Torsion” OR “Torsion Fracture”):ti,ab |
| #5 | (“Falls, Accidental” OR “Accidental Fall” OR “Fall, Accidental” OR “Falling” OR “Falls” OR “Slip and Fall” OR “Fall and Slip”) |
| #6 | (“Risk Factors” OR “Factor, Risk” OR “Risk Factor” OR “Population at Risk” OR “Populations at Risk” OR “Risk Scores” OR “Risk Score” OR “Score, Risk” OR “Risk Factor Scores” OR “Risk Factor Score” OR “Score, Risk Factor” OR “Health Correlates” OR “Correlates, Health” OR “Social Risk Factors” OR “Factor, Social Risk” OR “Factors, Social Risk” OR “Risk Factor, Social” OR “Risk Factors, Social” OR “Social Risk Factor”):ti,ab |
| #7 | #3 OR #4 OR #5 |
| #8 | #1 AND #2 AND #6 AND #7 |
| **CNKI** | |
| #1 | 主题 (糖尿病+Ⅱ型糖尿病+成人发病型糖尿病+非胰岛素依赖性糖尿病+非胰岛素依赖型糖尿病+二型糖尿病+2型糖尿病+1型糖尿病+Ⅰ型糖尿病) |
| #2 | 篇关摘 (2型糖尿病+Ⅱ型糖尿病+成人发病型糖尿病+非胰岛素依赖性糖尿病+非胰岛素依赖型糖尿病+二型糖尿病+1型糖尿病+Ⅰ型糖尿病) |
| #3 | 关键词 (2型糖尿病+Ⅱ型糖尿病+成人发病型糖尿病+非胰岛素依赖性糖尿病+非胰岛素依赖型糖尿病+二型糖尿病+1型糖尿病+Ⅰ型糖尿病) |
| #4 | #1 OR #2 OR #3 |
| #5 | 主题 (骨质疏松+骨质疏松症+骨质疏松骨折+摔倒+跌倒+骨折) |
| #6 | 篇关摘 (骨质疏松+骨质疏松症+骨质疏松骨折+摔倒+跌倒+骨折) |
| #7 | 关键词 (骨质疏松+骨质疏松症+骨质疏松骨折+摔倒+跌倒+骨折) |
| #8 | #5 OR #6 OR #7 |
| #9 | 主题 (危险因素+影响因素+预测因素) |
| #10 | 篇关摘(危险因素+影响因素+预测因素) |
| #11 | 关键词 (危险因素+影响因素+预测因素) |
| #12 | #9 OR #10 OR #11 |
| #13 | #4 AND #8 AND #12 |
| **WanFang** | |
| #1 | 主题 (糖尿病+Ⅱ型糖尿病+成人发病型糖尿病+非胰岛素依赖性糖尿病+非胰岛素依赖型糖尿病+二型糖尿病+2型糖尿病+1型糖尿病+Ⅰ型糖尿病) |
| #2 | 题名或关键词 (2型糖尿病+Ⅱ型糖尿病+成人发病型糖尿病+非胰岛素依赖性糖尿病+非胰岛素依赖型糖尿病+二型糖尿病+1型糖尿病+Ⅰ型糖尿病) |
| #3 | #1 OR #2 |
| #4 | 主题 (骨质疏松+骨质疏松症+骨质疏松骨折+摔倒+跌倒+骨折) |
| #5 | 题名或关键词 (骨质疏松+骨质疏松症+骨质疏松骨折+摔倒+跌倒+骨折) |
| #6 | #4 OR #5 |
| #7 | 主题 (危险因素+影响因素+预测因素) |
| #8 | 题名或关键词 (危险因素+影响因素+预测因素) |
| #9 | #7 OR #8 |
| #10 | #3 AND #6 AND #9 |
| **SinoMed** | |
| #1 | 主题词 (糖尿病+Ⅱ型糖尿病+成人发病型糖尿病+非胰岛素依赖性糖尿病+非胰岛素依赖型糖尿病+二型糖尿病+2型糖尿病+1型糖尿病+Ⅰ型糖尿病) |
| #2 | 关键词 (2型糖尿病+Ⅱ型糖尿病+成人发病型糖尿病+非胰岛素依赖性糖尿病+非胰岛素依赖型糖尿病+二型糖尿病+1型糖尿病+Ⅰ型糖尿病) |
| #3 | #1 OR #2 |
| #4 | 主题词 (骨质疏松+骨质疏松症+骨质疏松骨折+摔倒+跌倒+骨折) |
| #5 | 关键词 (骨质疏松+骨质疏松症+骨质疏松骨折+摔倒+跌倒+骨折) |
| #6 | #4 OR #5 |
| #7 | 主题词 (危险因素+影响因素+预测因素) |
| #8 | 关键词 (危险因素+影响因素+预测因素) |
| #9 | #7 OR #8 |
| #10 | #3 AND #6 AND #9 |
