# Supplementary material for: Risk factors for osteoporosis in elderly patients with type 2 diabetes: A protocol for systematic review and meta-analysis
Source: PLoS One. 2025 Feb 27;20(2):e0319602. doi: 10.1371/journal.pone.0319602 (PMC11867315; doi:10.1371/journal.pone.0319602)
Supplement: Attachment — Submitted filename: Review 31.pdf [file pone.0319602.s006.pdf]

Title: Risk factors for osteoporosis in elderly patients with type 2 diabetes: a protocol for systematic review and meta-analysis

Journal: PLOS NOE

Reviewer: Seyed Amirhossein Mazhari

Reviewer's report:

This structured manuscript provides a comprehensive framework for systematically reviewing and analyzing risk factors associated with osteoporosis in elderly patients with type 2 diabetes.

The manuscript is analyzed appropriately and provides suitable results. Additionally, adherence to multiple guidelines, ensures transparency and accuracy in the methodology. The Discussion section need to be more vigorously approached and a detailed explanation into the issue is required.

At the end I have some questions about this article.

The specific questions and recommendations are as follows:

1:
Although the Introduction section offers a suitable framework, it is lacking in details and would benefit from being expanded. It is advised to include more references in this area. The primary text's conclusion also needs to be extended and revised. Think about including additional evidence.

2:
After the Discussion section, I suggest adding a new section regarding limitations and adding additional details about them, along with suggestions for how to resolve these problems in current and future research. A more thorough description would be helpful.

3:
It is advised that a new section explaining any acronyms used in the article be added at the end, before the references.

4:
There is no Conclusion in the manuscript. The authors are advised to create a dedicated section for concluding points at the end of the article.

5:
The discussion should be more comprehensive. It should be more vigorously approached and a detailed explanation into the issue is required. Adding more references is suggested.

6:
The manuscript is generally clear and concise; however, minor grammatical adjustments could enhance readability.

Thanks to the authors for their hard work on this Study. It’s a pleasure to review such a contribution to the field.
