# Supplementary material for: Risk factors for osteoporosis in elderly patients with type 2 diabetes: A protocol for systematic review and meta-analysis
Source: PLoS One. 2025 Feb 27;20(2):e0319602. doi: 10.1371/journal.pone.0319602 (PMC11867315; doi:10.1371/journal.pone.0319602)
Supplement: Attachment — Submitted filename: response to reviewers.docx [file pone.0319602.s009.docx]

Dear editor and reviewers:

On behalf of my co-authors, we appreciate editor and reviewers very much for their constructive comments and suggestions on our manuscript entitled *“Risk factors for osteoporosis in elderly patients with type 2 diabetes: a protocol for systematic review and meta-analysis”*

We have studied the comments carefully and made corrections which we hope meet with approval. The corrections are in the manuscript and the responds to the reviewers’ comments are as follows**:**

**Reviewer #1:**

(1) Line 112: it is suggested to add your reference for this classification.

Thank you for your kind advice. We added the reference of the classification.

(2) It is suggested to include data regarding the medications of patients and adjustments in the statistical analysis as well.

Thank you for your kind advice. We added the medications of patients and adjustments in the statistical analysis. Here are our corrections:

*“High heterogeneity will be examined utilizing Stata 16.0 software, focusing on age, gender, geography, sample size, medication (dosage, type of medication), treatment duration, and various risk factors via subgroup or sensitivity analysis. If heterogeneity exceeds 75%, a meta-analysis will not be performed. We shall employ descriptive analysis.”*

(3) Data extraction part is somehow general and needs to be more consice.

Thank you for your very kind advice. We rewrote our data extraction part. It’s more concise than before.

*“Two researchers (W.H. and Z.Y) independently screen literature and conduct cross checking. When there is a disagreement, the final decision is made by the third researcher (Y.R.). Extract data using a pre-designed data table by the researcher, which mainly includes the following information: author, publication year, study type, study location, country, sample size, gender, age, treatment medication, medication adjustments, osteoporosis related influencing factors, OR values and 95% CI of risk factors, etc. If information is missing, we will contact the first author or corresponding author every Monday and Friday for two consecutive months. If no response is received, the study will be included in the research, but only a narrative description will be provided.”*

**Reviewer #2:**

(1) Although the Introduction section offers a suitable framework, it is lacking in details and would benefit from being expanded. It is advised to include more references in this area. The primary text's conclusion also needs to be extended and revised. Think about including additional evidence.

Thank you for your suggestion. We corrected the introduction part.

*“Diabetes is a prevalent chronic non-communicable disease, which has become a serious public health problem in the world. Around 537 million persons are impacted by diabetes[1]. By 2030, this figure is anticipated to rise to 643 million. Diabetes has resulted in economic losses of no less than 966 billion US dollars, a fourfold increase since 2009[2]. The prolonged abnormal blood glucose levels in diabetes can lead to microvascular complications (such as diabetic retinopathy and diabetic nephropathy), macrovascular complications (such as coronary artery disease, heart failure, and peripheral arterial disease), neuropathy, and diabetic foot, as well as disrupt bone metabolism and induce osteoporosis.[3].*

*The incidence of osteoporosis in diabetes patients is increasing alongside the progression of diabetes[4]. Osteoporosis is a chronic metabolic bone disorder marked by reduced bone mass and microstructural deterioration of bone tissue, resulting in heightened bone fragility and an elevated risk of fractures[5]. Abnormal bone metabolism in diabetic patients can diminish bone density and compromise bone structure[6], with osteoporosis prevalence reaching 27.67%[6], The risk of osteoporosis in type 2 diabetes patients is significantly higher than that of ordinary people. Research shows that the risk of fracture healing damage in diabetes patients is 2.11 times that in non-diabetes patients [7]. Diabetes increases the fracture risk by destroying bone metabolism and accumulating advanced glycation end products (AGEs), further exacerbating bone fragility[8]. Moreover, the risk of osteoporosis increases with the prolongation of the course of diabetes [9] and leads to fractures, falls, brain injuries, and other adverse consequences [10].*

*In addition, the risk of osteoporosis in elderly diabetes patients is significantly higher than other diabetes patients. On the one hand, the physical function of elderly people degenerates with age, and calcium content in bones decreases, resulting in osteoporosis; On the other hand, elderly patients with diabetes are more likely to suffer from sleep disorders, which are closely related to low bone density and osteoporosis [11]. Sleep disorders can inhibit the function of osteoblasts, promote bone absorption and decomposition, weaken the ability of bone microstructure damage repair accumulated with age, and lead to a higher risk of osteoporosis [12]. Long-term diabetes impairs renal function, resulting in diminished synthesis of vitamin D, which subsequently affects intestinal calcium absorption [13]. Insulin regulates osteoblasts; however, in diabetic individuals, inadequate insulin results in impaired osteogenesis and insufficient bone production [14]. All the aforementioned factors cause individuals with diabetes more susceptible to osteoporosis.*

*Though diabetic osteoporosis carries serious effects, it has not attracted much attention. The Asia Pacific Osteoporosis Association (APCO) indicates that merely 31% of diabetic patients have undergone bone health evaluations[3]. Moreover, the bone mineral density (BMD) of individuals with type 2 diabetes is greater than that of age-matched non-diabetic individuals[9, 15], however, BMD does not correlate directly with bone strength. Consequently, individuals may undervalue the risk of osteoporosis in diabetic patients.*

*Several hypoglycemia therapeutic interventions could influence bone metabolism[16], and certain diabetic medications may expedite the progression of osteoporosis. Thiazolidinedione (TZD) could elevate the risk of weight gain, edema, fractures, and heart failure in diabetic patients [17]. Sodium-glucose cotransporter-2 inhibitors (SGT2I) may influence bone resorption by altering electrolyte homeostasis, calcium and phosphorus metabolism, while also elevating parathyroid hormone levels and diminishing 1,25-(OH)-vitamin D concentrations[18]. Because diabetes is a long-term disease that affects the whole body, it usually takes longer for people with diabetes to heal from fractures. This makes them more likely to get hospital-acquired infections, muscle loss, and pneumonia[19].*

*The accumulation of the receptor of advanced glycation endproducts (RAGE) in the cortical bone of diabetes patients will harden type I collagen in the bone matrix, reduce bone strength, increase bone fragility, and promote osteoblast apoptosis [20]. In addition, the course of diabetes[21], insulin resistance[22], bone marrow fat accumulation, and low bone turnover level are all factors that cause the increased risk of fracture in diabetes patients[20]. Therefore, people may underestimate the risk of osteoporosis in patients with diabetes.*

*Although it is known that abnormal blood glucose in diabetes can cause osteoporosis, the pathogenesis and influencing factors of osteoporosis in type 2 diabetes are still unclear. There are few studies on osteoporosis in elderly patients with type 2 diabetes, and the risk factors pointed out in various studies are different [23-27]. Therefore, the purpose of this study is to identify the risk factors of DOP through meta-analysis, to help medical staff identify high-risk groups in time and take effective intervention strategies.”*

(2) After the Discussion section, I suggest adding a new section regarding limitations and adding additional details about them, along with suggestions for how to resolve these problems in current and future research. A more thorough description would be helpful.

Thank you for your good advice. We created the limitation part with its solutions. Here are our corrections.

*“This study may have some potential limitations. (1) We plan to only include observational studies, which may not cover all the influencing factors of DOP and can only obtain modifiable risk and protective factors that exist in the existing evidence base. (2) This study will be limited to Chinese and English studies and may overlook important research in other languages. However, considering that team members are proficient in both Chinese and English, using translation software to translate other languages may result in translation errors, leading to significant deviations in the results. (3) This article only focuses on osteoporosis in elderly patients with diabetes. The osteoporosis situation of young diabetes patients and postmenopausal female diabetes patients is also not optimistic. In the future, research can be carried out on this kind of population, and the common influencing factors of osteoporosis in young and old diabetes patients may be found. (4) Since we will include all the studies on risk factors of osteoporosis in diabetes patients, the number of literature on some influencing factors may be small, so we will use the form of table and description to elaborate, and the research results may also have high heterogeneity, so we will use subgroup analysis and sensitivity analysis to interpret the heterogeneity, and use narrative description to interpret some results. (5) To include as much literature as possible for analysis, we will also search for grey literature. However, grey literature may have lower quality and affect the outcome, so we will only include grey literature with higher research quality.”*

(3) The references. It is advised that a new section explaining any acronyms used in the article be added at the end, before

Thank you for your detailed advice. We added the abbreviation part at the end. Here are our corrections.

*“DOP: Diabetic Osteoporosis*

*NOS: the Newcastle Ottawa Quality Assessment Instrument*

*GRADE: Grading of Recommendations, Assessment, Development, and Evaluation*

*APCO: The Asia Pacific Osteoporosis Association*

*BMD: The Bone Mineral Density*

*SGT2I: Sodium-Glucose Cotransporter-2 Inhibitors*

*PRISMA-P: Preferred Reporting Items for Systematic reviews and Meta-Analyses*

*MOOSE: Meta Analysis of Epidemiological Observational Studies*

*BMI: Body Mass Index*

*OR: Odd Ratio*

*CI: Confidence Interval*

*SMD: Standard Mean Deviation*

*WMD: Weighted Mean Deviation”*

(4) There is no Conclusion in the manuscript. The authors are advised to create a dedicated section for concluding points at the end of the article.

Thank you for your good advice. We added the conclusion part at the end. Here are our corrections.

*“This study will strictly follow various guidelines (including PRISMA, NOS, MOOSE, GRADE) to ensure that our research complies with reporting standards. Searching 8 databases in both Chinese and English can obtain DOP epidemiological data from different cultural backgrounds. This study will explore the heterogeneity found under different age, gender, region, sample size, treatment drugs (drug dosage, drug type), treatment time, and different types of influencing factors. In addition, identifying the risk and protective factors of DOP will enhance our understanding of DOP patients. This study aims to explore the modifiable risk factors and potential protective factors in DOP patients, which is of great significance for clinical practice. The results of the study may help to develop a health promotion and disease prevention plan for type 2 diabetes aimed at reducing DOP.”*

(5) The discussion should be more comprehensive. It should be more vigorously approached and a detailed explanation into the issue is required. Adding more references is suggested.

Thank you for your good advice. We rewrote the discussion part. Here are our corrections. Here is our discussion part with more references.

*“Currently, there is no comprehensive assessment to thoroughly analyze the risk factors of osteoporosis diabetic patients. This study will be the first comprehensive investigation into the risk factors for DOP. This review's findings may enhance health outcomes for older diabetes patients by facilitating early identification and strengthening the surveillance or management of diabetic osteoporosis, hence mitigating deteriorating risk factors.*

*Diabetes is a chronic endocrine metabolic disorder, and prolonged hyperglycemia[32] and oxidative stress[33] will lead to osteoporosis in diabetic patients. Secondly, with advancing age, variables such as diminished sex hormone levels[34] and insufficient physical activity elevate the risk of osteoporosis in elders. Factors including diminished digestive function, impaired movement, and reduced vision in the elderly exacerbate their vulnerability to fractures following osteoporosis[35]. Moreover, individuals with diabetes frequently exhibit obesity and hyperlipidemia, and data suggests that this correlates with elevated bone density and diminished bone strength[36, 37]. Although the bone mineral density of diabetes patients is normal or higher, the fracture risk of diabetes patients increases by 40-70% [15, 38].*

*Consequently, diabetic patients tend to get fractures despite elevated bone density levels[15]. The chronic complications of diabetes, including diabetic nephropathy[39], diabetic liver disease[40], and diabetic retinopathy[41], along with the coexistence of multiple chronic conditions such as hypertension, coronary heart disease, and heart failure, hinder the clinical management of elderly diabetic patients and may elevate their mortality risk[42]. Some studies indicate that the risk of hip fracture in diabetic patients is 1.7 times more than that of the general population, and the mortality rate following hip fracture is similarly elevated compared to non-diabetic individuals[43]. Many studies have investigated diverse risk factors associated with osteoporosis in patients with diabetes. The determinants encompass age, gender[44, 45], pharmacological agents (such as thiazolidinediones)[46], comorbidities (including diabetic nephropathy[47], hypertension[48]), and the disease's severity. Certain medications not only effectively regulate blood glucose levels but also contribute positively to the prevention of osteoporosis, including metformin[49] and dipeptidyl peptidase-4 inhibitors[50]. The enduring protective efficacy of the aforementioned medications on bone health requires more validation. Upon meticulous analysis of the available data, we detected deficiencies in prior research. Numerous research primarily investigates the risk factors, prevention, and treatment of osteoporosis in the aged population. There exists a distinction between the osteoporosis observed in senior diabetic patients and that in the general elderly population. The habitual application of X-ray for assessing the risk of osteoporosis does not reliably indicate the risk of osteoporosis or fractures in older individuals with diabetes. Diabetes significantly affects bone density, bone matrix, bone structure, and bone metabolism[51]. X-rays alone are insufficient for making precise assessments. Consequently, it is imperative to conduct a thorough evaluation of the risk factors for osteoporosis in older individuals with diabetes to identify all pertinent risk variables and establish appropriate preventative or treatment measures.”*

(6) The manuscript is generally clear and concise; however, minor grammatical adjustments could enhance readability.

Thank you for your concerned advice. We carefully checked our manuscript and rewrote many sentences in quillbot. Maybe some words or sentences may not be very authentic, but we believe it’s an academic language. Greatly appreciate your notice that some words and sentences have some grammatical mistakes.
