# Supplementary material for: Risk factors for osteoporosis in elderly patients with type 2 diabetes: A protocol for systematic review and meta-analysis
Source: PLoS One. 2025 Feb 27;20(2):e0319602. doi: 10.1371/journal.pone.0319602 (PMC11867315; doi:10.1371/journal.pone.0319602)
Supplement: Attachment — Submitted filename: Response_to_Reviewers_auresp_2.docx [file pone.0319602.s010.docx]

Dear editor and reviewers:

On behalf of my co-authors, we appreciate editor and reviewers very much for their constructive comments and suggestions on our manuscript entitled *“Risk factors for osteoporosis in elderly patients with type 2 diabetes: a protocol for systematic review and meta-analysis”*

We have studied the comments carefully and made corrections which we hope meet with approval. The corrections are in the manuscript and the responds to the reviewers’ comments are as follows**:**

**Reviewer #1:**

(1) The authors have addressed my comments, completely. I have no further comments for them. I only suggest to make all data available as there is no restrictions for using data from already published papers.

Thank you for your kind advice. We have added all guideline data that we are going to use. Here shows the extra guideline files we added.

*“****Supporting information***

*S2 File. MOOSE (Meta-analyses Of Observational Studies in Epidemiology) Checklist.*

*(PDF)*

*S4 File. The Newcastle Ottawa Quality Assessment Instrument (NOS).*

*(DOCX)”*

**Reviewer #2:**

1:

Although the Introduction section offers a suitable framework, it is lacking in details and would benefit from being expanded. It is advised to include more references in this area. The primary text's conclusion also needs to be extended and revised. Think about including additional evidence.

Appropriately corrected, both introduction and conclusion are now informative.

Thank you for your kind advice. We are happy to know it’s appropriate corrected.

2:

After the Discussion section, I suggest adding a new section regarding limitations and adding additional details about them, along with suggestions for how to resolve these problems in current and future research. A more thorough description would be helpful.

A new section has been created and limitations are explained in the dedicated section.

Thank you for your useful advice. It’s more integrative with this limitation section.

3:

It is advised that a new section explaining any acronyms used in the article be added at the end, before the references.

New section has been incorporated into the text regarding any acronym used in the main text.

Thank you for your kind advice. It’s more integrative with acronym section.

4:

There is no Conclusion in the manuscript. The authors are advised to create a dedicated section for concluding points at the end of the article.

The structure of the text is now more standardized and conclusions are being discussed at the end of the manuscript.

Thank you for your very useful suggestion. It’s better with a conclusion in this protocol article.

5:

The discussion should be more comprehensive. It should be more vigorously approached and a detailed explanation into the issue is required. Adding more references is suggested.

Appropriately addressed.

Thank you for your pretty constructive suggestion. The discussion part is more logical following your advice. It’s much better than before.

6:

The manuscript is generally clear and concise; however, minor grammatical adjustments could enhance readability.

Overall language and readability are improved and the text grammar has been enhanced.

Thank you for your very careful advice. You are right, some sentences are inappropriate, so we rewrote and corrected our language.

Finally, we pretty appreciate two reviewers’ very detailed comments. Thank you for your very helpful and hard work to review our article. We would like to make this article better if there are more points that you think we need to make a change. Thanks again.
